# Genome-Wide Analysis Identifies *ScTCP6* as a Stress Responsive Gene in Rye

**DOI:** 10.3390/cimb48030266

**Published:** 2026-03-02

**Authors:** Yanyan Ren, Rui Ma, Zhiruo Wang, Ling Li, Muhua Xie, Tingting Jiang, Jing Zhang, Qinggui Lian

**Affiliations:** 1 College of Agriculture, Shihezi University, Shihezi 832003, China; renyanyan2025@163.com; 2 College of Plant Protection, Northwest A&F University, Yangling 712100, China; nwafumary@nwafu.edu.cn (R.M.); 13453014628@163.com (M.X.); tingtingjiang@nwafu.edu.cn (T.J.); 3 Chemicals, Minerals & Metallic Materials Inspection Centre of Tianjin Customs, Tianjin 300450, China; wodeyouxiang622@163.com; 4 Bayannur City Modern Agriculture and Animal Husbandry Development Center, Bayannur 015000, China; 18204730431@163.com; 5 Sanya Nanfan Research Institute, School of Tropical Agriculture and Forestry, Hainan University, Haikou 570228, China

**Keywords:** abiotic stress, evolution, hormones, segmental duplication, *TCP* gene family

## Abstract

Teosinte branched1/cycloidea/proliferating cell factor (TCP) transcription factors are key regulators of plant growth and stress adaptation. However, their evolutionary history and functional divergence in rye (*Secale cereale* L.) remain unclear. Here, 26 *ScTCP* genes were identified from the reference rye genome. Phylogenetic and collinearity analyses with six representative cereals (*Secale cereale*, *H. vulgare*, *O. sativa*, *T. aestivum*, *Z. mays*, and *A. tauschii Coss*) revealed that segmental duplication, rather than tandem repetition, drove *ScTCP* expansion, with *ScTCP2* located in a conserved syntenic block shared across the *Poaceae* family. Promoter analysis identified numerous hormone- and stress-responsive *cis*-elements, while a predicted protein–protein interaction network indicated extensive cross-talk with ERF and MYB transcription factors. Expression profiling of 12 representative *ScTCP* genes using qRT-PCR across different organs, developmental stages, six abiotic stress conditions, and three hormone treatments showed that *ScTCP6* plays an important role in rye development and in responses to hormonal signals and abiotic stresses. Therefore, this study provides the first genome-wide characterization of the *TCP* gene family in rye and contributes to a broader understanding of the evolution and functional diversification of the *TCP* superfamily in higher plants.

## 1. Introduction

Rye (*Secale cereal* L.), a diploid member of the *Triticeae* [1,2], is characterized by a large and complex 7.86 G genome [3] and a close genetic relationship with wheat (*Triticum aestivum* L.) and barley (*Hordeum vulgare* L.) [4,5]. Owing to its remarkable tolerance to of poor soils, cold, drought and salinity, rye is increasingly cultivated on marginal lands and is valued as a reservoir of stress-adaptation genes for cereal improvement [6,7]. The recent release of a high-quality rye genome assembly has accelerated gene discovery and functional studies, establishing a foundation for targeted molecular breeding [8,9].

In plants, gene-expression networks are orchestrated by transcription factors (TFs), which bind *cis*-regulatory elements and recruit co-activators or repressors [10,11]. Among these, the TCP Teosinte branched1, Cycloidea, and Proliferating cell factor family is defined by a conserved basic helix-loop-helix (bHLH) domain that mediates DNA binding [12]. Plant TCPs are divided into class I and class II, with the latter further separated into the CIN and ECE (CYC/TB1) clades [12,13,14]. *TCP* genes have been cataloged in various plant species through comparative genomics, including *Arabidopsis thaliana* [15], *Oryza sativa* L. [15], *Broussonetia papyrifera L.* [16], *Vitis vinifera* L. [17], *Zea mays* L. [18], *Solanum lycopersicum* [19], *Malus domestica* [20], *Hordeum vulgare* L. [21]. *Panicum virgatum* L. [22], *Triticum aestivum* L. [23], and *Prunus mume* [24]. However, research on *TCP* transcription factors in *rye* remains limited. TCP transcription factors have been demonstrated to regulate diverse plant developmental processes [25,26]. For instance, tillering in rice is modulated by *OsTB1* [27,28], and drought resistance in Arabidopsis is enhanced by *AtTCP20* through the mediation of stomatal closure [29]. In wheat, *TaTCP21*-A negatively regulates cold tolerance by repressing the expression of the cold-responsive gene *TaDREB1C* [30]. In barley, the TCP transcription factor HvTB2 forms a heterodimer with VRS5, thereby regulating spike architecture [31]. Furthermore, overexpressing *OsTCP19* reportedly activates the IAA, JA, and ABA signaling pathways, improving abiotic stress resistance [32]. Despite this extensive characterization in other plants, systematic information on *TCP* genes in rye is scarce.

Based on the latest published whole genome sequence of rye, this study performs a systematic, genome-wide analysis of the *ScTCP* gene family, with the aim of elucidating its physiological functions and evolutionary relationships in gramineous crops and establishing a theoretical basis for crop genetic improvement. This study included (i) the identification of 26 *TCP* genes and determination of their chromosomal distribution, gene structures, conserved motifs, and phylogenetic relationships; (ii) the dissection of duplication patterns and cross-species synteny to infer evolutionary trajectories; (iii) the mining of promoter regions for *cis*-acting elements and construction of a putative protein-interaction network; and (iv) profiling of representative *ScTCP* expression across organs, fruit-development stages, and multiple hormone or abiotic treatments. These results provide a theoretical basis for understanding the *ScTCP* gene family and nominate priority candidates, such as *ScTCP6*, for functional validation and molecular breeding aimed at improving rye architecture and stress resilience.

## 2. Materials and Methods

### 2.1. Gene Identification

Rye (*Secale cereale*) data were retrieved from Ensembl (http://ensemblgenomes.org), and the three-helix structures of all TCP proteins in the *Arabidopsis* were obtained. The rye genome was initially screened using BLASTp 2.15.0+ with an identity cutoff of ≥100 and an e-value ≤ 1 × 10^−10^. The TCP conserved domain was then downloaded from the Pfam database (http://pfam.xfam.org, accessed on 8 June 2024) to identify all *TCP* genes in rye using HMMER 3.0 at 0.01 HMM model truncation; (http://plants.ensembl.org/hmmer/index.html, accessed on 8 June 2024) [33]. The 26 identified TCP proteins were verified using PFAM and SMART (http://smart.emblheidelberg.de, accessed on 7 October 2024) to ascertain the presence of the bHLH domain, and then used as initial sequences to confirm TCP proteins (https://blast.ncbi.nlm.nih.gov/Blast.cgi?PROGRAM=blastp&PAGE_TYPE=BlastSearch&LINK_LOC=blasthome, accessed on 8 November 2024) with BLASTp. to further verify the results. ExPasy (https://web.expasy.org/compute_pi/, accessed on 1 December 2024) was used to characterize their features. Cis-acting element analysis and protein interaction prediction were performed using PlantCare (http://bioinformatics.psb.ugent.be/webtools/plantcare/html, accessed on 12 December 2024) and PlantTFDB [34].

### 2.2. TCP Phylogenetic Analysis, and Gene Duplication Events

The *A. thaliana* TCP (AtTCP) protein sequences were aligned with ScTCP sequences using multiple sequence alignment (MSA) and refined manually with GeneDoc and MEGA6.0 software [35]. Exon–intron structures were analyzed using the Gene Structure Display Server (GSDS, http://gsds.cbi.pku.edu.cn), and conserved motifs were identified using the MEME (https://meme-suite.org/meme/, accessed on 1 January 2025). The Circos program was used to map all *ScTCP* genes onto the seven *rye* chromosomes, and the multicollinearity scanning toolkit (MCScanX) was applied to determine collinearity and gene duplication events. Finally, Dual Synteny Plotter (https://github.com/CJ-Chen/TBtools, accessed on 15 January 2025) was used to assess homology among different species.

*ScTCP* genes from *T. aestivum*, *H. vulgare*, *O. sativa*, *A. thaliana*, *Z. mays*, and *A. tauschii* Coss were obtained from UniProt (https://www.uniprot.org/) and aligned using MEGA 6.0. The neighbor-joining (NJ) tree was constructed using the Jukes–Cantor model with 1000 bootstrap replicates.

### 2.3. Plant Materials, Growth Conditions, and Different Abiotic Stress in Rye

We gratefully acknowledge Prof. Cheng Jianping (Guizhou University) for providing rye seeds (Weining variety) used in this experiment. The seeds were cultivated in pots containing a 1:1 mixture of soil and vermiculite under controlled growth conditions: 16 h light at 25 °C, 8 h dark at 20 °C, and 75% relative humidity. Stems, roots, leaves, fruits, anthers, and styles were collected from five healthy plants grown under identical conditions. All samples were immediately frozen in liquid nitrogen for later use. At the seedling stage (21 days after germination), plants were subjected to various abiotic stresses: salt (5% NaCl), water immersion (whole plant), drought (30% PEG 6000), ultraviolet radiation (70 W/cm^2^, 220 V, 30 W), high temperature (40 °C), and low temperature (4 °C). Each treatment was performed in five replicates. qRT-PCR analysis was conducted 1, 4, and 12 h after treatment to examine expression patterns of 21 additional *TCP* genes under different stresses. At the same growth stage (21 days after germination), plants were also treated with ABA (100 μmol/L), IAA (100 μmol/L), and GA_3_ (100 μmol/L). Fruits were first collected at the onset of the filling stage, followed by collections at 7, 14, 21, 28, and 35 days. Each treatment included five replicates to ensure data accuracy and reliability. Meanwhile, these samples were performed by qRT-PCR with at least three technical repeats.

### 2.4. Total RNA Extraction, cDNA Reverse Transcription, and qRT-PCR Analysis

Total RNA was extracted from all *rye* samples using the Plant RNA Extraction Kit (Tiangen Biochemical Technology Co., Ltd., Beijing China), and first-strand cDNA was synthesized from 1 µg of total RNA using HiScript III R RT SuperMix (+ gDNA wiper) for qRT-PCR (Vazyme Biotech Co., Ltd., Nanjing, China). For *TCP* gene expression analysis, qRT-PCR was performed using five independent biological replicates. Primers were designed with Beacon Designer 7 (Supplementary Table S6) [36], and actin (GADPH) served as the reference gene. Real-time qPCR reaction (ChamQ Universal SYBR qPCR Master Mix-Q711., Ltd., Nanjing, China) included 40 cycles with parameter settings as follows: pre-denaturation at 95 °C for 30 s, denaturation at 95 °C for 5 s, annealing at 60 °C for 20 s, and extension at 72 °C for 20 s. Relative mRNA expression levels were calculated using the 2^−ΔΔCt^ method [37]. Genes showing ≥ 2-fold or ≤0.5-fold expression (treatment/control) were considered significantly upregulated or downregulated.

### 2.5. Statistical Analyses

JMP 6.0 (SAS Institute, Minato City, Japan) software was used to perform analysis of variance (ANOVA) [38]. For multiple comparison tests, significance levels of *p* < 0.05 and *p* < 0.01 were applied using the least significant difference (LSD) test. Finally, Origin 8.0 (OriginLab, Northampton, MA, USA) was used to generate the histogram [39].

## 3. Results

### 3.1. Conserved Motif and Structure Analysis of ScTCP Genes

**Figure 1:**
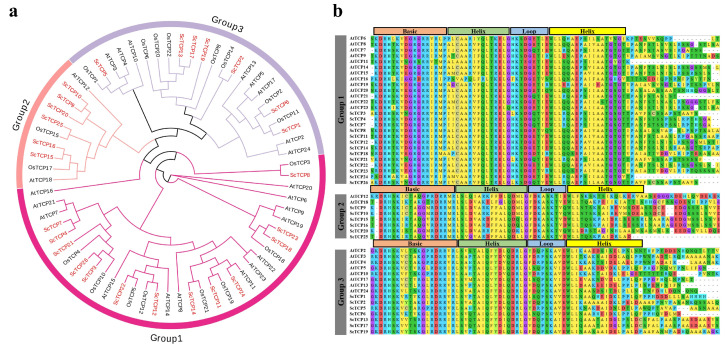
The evolutionary relationship and sequence alignment of the rye TCP proteins. (**a**) Rootless phylogenetic tree of 26 *ScTCP* genes from *rye*, *Arabidopsis thaliana*, and *rice*. Rye *TCP* genes are shown in red, and *Arabidopsis* and *rice TCP* genes are shown in black. (**b**) Multiple sequence alignment of bHLH domains among the three subfamilies.

In this study, 26 *TCP* genes were identified and characterized from the rye genome and were designated *ScTCP1* through *ScTCP26*. This number is comparable to the 24 *AtTCP* genes and 22 *OsTCP* genes previously identified in *Arabidopsis* and rice, respectively. Using the Cenci and Rouard method, the *ScTCP* genes were classified into three subfamilies (Figure 1a). Subfamily 1 contained the largest number of members (13 *ScTCPs*), whereas subfamily 2 contained the fewest members (6 *ScTCPs*). Phylogenetic analysis showed that *ScTCPs* clustered closely with *OsTCPs* and *AtTCPs*, with bootstrap support ≥ 70, suggesting that TCP proteins in rye and rice are likely homologous and may possess similar biological functions. All *ScTCP* genes were found to possess BHLH (basic, helix, loop, helix) conserved domains of approximately 100 amino acid residues, including 12 conserved amino acids (Figure 1b). However, structural variation was observed; the conserved domains of subfamilies 2 and 3 contain four additional amino acids compared to subfamily 1. Overall, the conserved domains of *ScTCP* genes demonstrate a tendency towards evolutionary conservation.

The structural diversity and intron distribution of *TCP* genes in *rye* were analyzed using DNA sequences. Genes belonging to the same subfamily were found to exhibit similar exon-intron structures, which is attributed to their functions and evolutionary relationships (Figure S2a,b). All ScTCPs contain Motif 1 and Motif 3, suggesting that these motifs play an important role in maintaining *ScTCP* functions.

### 3.2. Evolutionary Analysis of the ScTCP Genes and TCP Genes of Different Species

**Figure 2:**
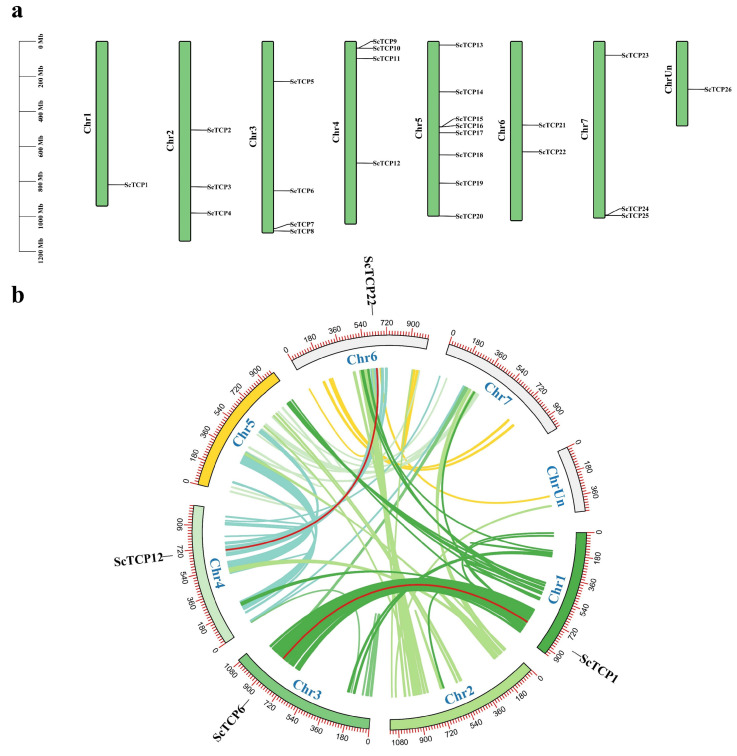
Chromosome distribution and synteny blocks of *TCP* genes in rye. (**a**) Distribution of 26 *ScTCP* genes across seven chromosomes. Green bars represent chromosomes with their respective numbers, and the left scale indicates chromosome length. (**b**) Schematic diagram illustrating the chromosome distribution and interchromosomal relationships of *TCP* genes in rye. Colored lines indicate all rye synteny blocks, and red lines represent duplicated *TCP* gene pairs.

Based on the rye genome information, the chromosomal positions of the *ScTCP* genes were plotted. A total of 25 *ScTCP* genes were found to be distributed unevenly across seven chromosomes (Figure 2a, Table S1), while *ScTCP26* was localized to an unknown chromosome. Chr5 contained the highest number of *ScTCP* genes (8, ~30.7%), followed by Chr3 and Chr4, which each harbored the same number of genes (25.8%), whereas Chr1 contained only a single gene (*ScTCP1*).

Gene duplication events showed the evolutionary regulation of gene families [40]. Tandem repeats and segment duplications are known to influence the evolution, structure, and function of gene families [41,42]. In this study, the *ScTCP* gene family was shown to lack tandem duplication events but was found to contain two pairs of segmental duplication events. These four segments form two fragment replicates located on chromosomes LG1, LG3, LG4, and LG6 (Figure 2b, Table S2).

**Figure 3:**
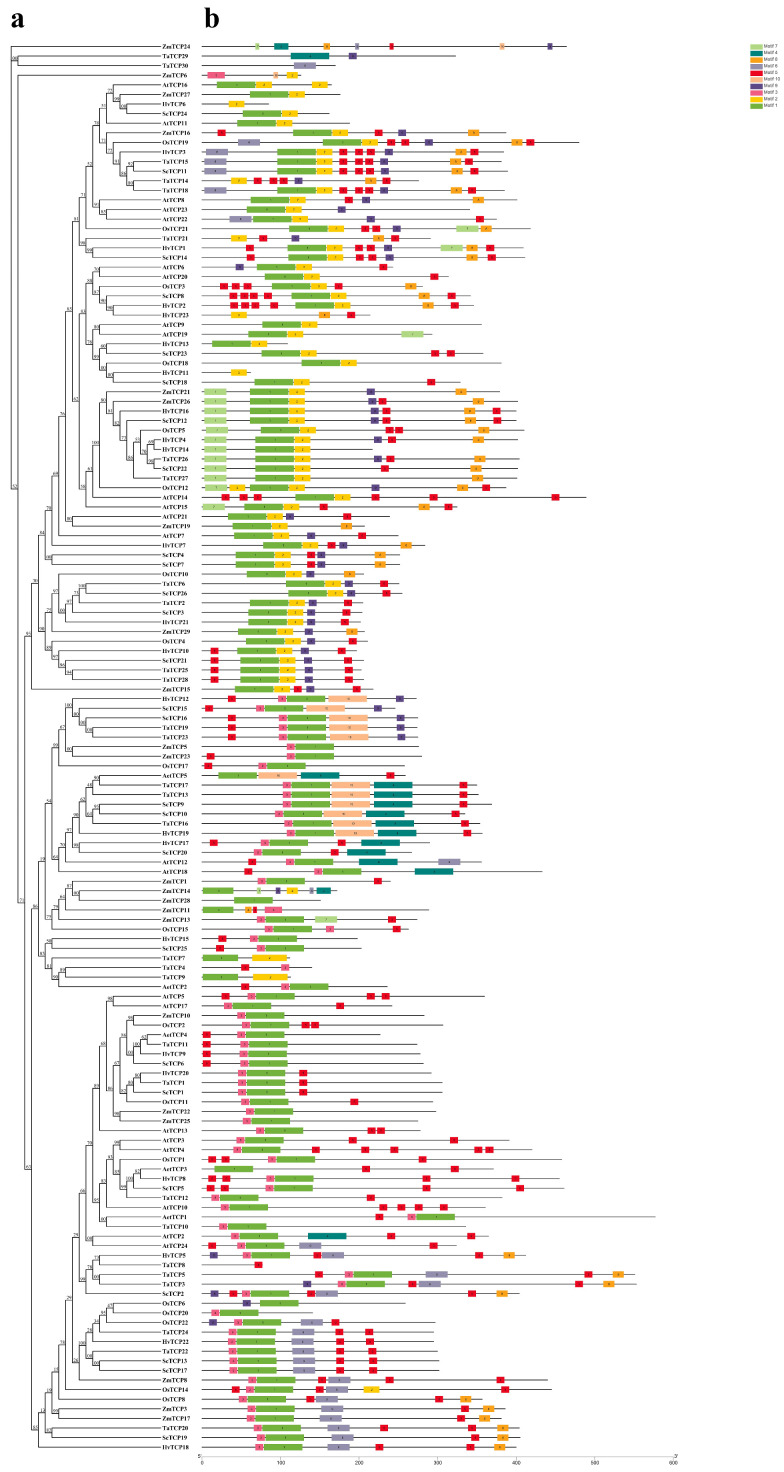
Analysis of conserved motifs and gene structures of 26 *ScTCP* genes based on phylogenetic relationships. (**a**) A phylogenetic tree was constructed using the amino acid sequences of rye *TCP* genes with the NJ method. (**b**) Ten conserved motifs predicted in TCP proteins are displayed as boxes in different colors.

To explore the evolutionary mechanisms of the rye TCP family, a comparative analysis was conducted with the classic plant *A. thaliana* and five monocotyledonous plants (*O. sativa*, *Z. mays*, *T. aestivum*, *H. vulgare*, and *A. tauschii*) closely related to *rye*. Different degrees of relatedness were observed between rye *ScTCP* and the *TCPs* of *A. tauschii* (5), *H. vulgare* (20), *O. sativa* (22), *A. thaliana* (24), *Z. mays* (29), and *T. aestivum* (66). In general, wheat *TCP* genes were found to be highly homologous with *rye ScTCP*. Ten significant motifs were identified in rye (Figure 3, Table S3). Almost all *ScTCP* genes contain Motif 1, while Motif 2 is exclusively present in subfamily 1. Motif 5, the most abundant, exhibits numerous repetitions and an uneven distribution. In contrast, Motif 9 is found only in subfamilies 1 and 2.

**Figure 4:**
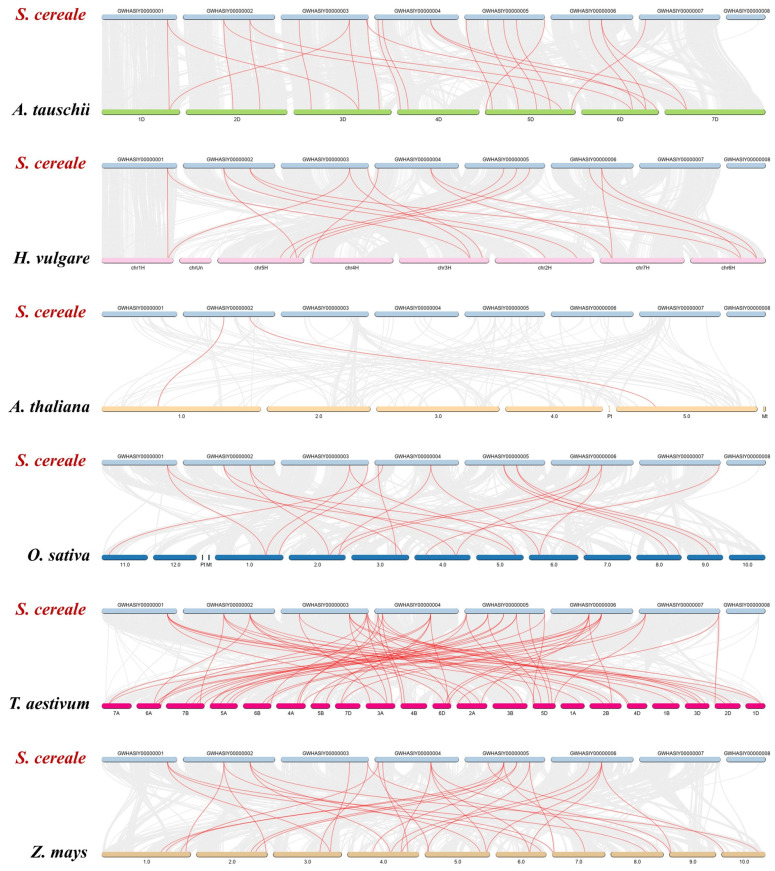
Homology between *rye* and six representative plant species (*Secale cereale*, *A. thaliana*, *H. vulgare*, *O. sativa*, *T. aestivum*, *Z. mays*, and *A. tauschii Coss*). Red lines denote collinear *TCP* gene pairs between *rye* and other species.

A collinearity analysis of *rye TCP* genes with six other species revealed varying numbers of collinear gene pairs among them. The highest collinearity was recorded with wheat (66 pairs), while the lowest was found in *A. thaliana* (2 pairs). Several collinear gene relationships were conserved; for instance, *ScTCP2* was found to be collinear with *HORVU5Hr1G103400.1*/*TraesARI2A01G274900.1*/*Os07t0152000-01*/*AET2Gv20566900.3/AT1G30210.1*/*Zm00001d007868_T001*. Furthermore, seven *ScTCP* genes (*ScTCP1*, *ScTCP6*, *ScTCP8*, *ScTCP12*, *ScTCP15*, *ScTCP21*, and *ScTCP22*) were identified as collinear with genes in *H. vulgare*, *A. tauschii*, *O. sativa*, *Z. mays*, and *T. aestivum*, suggesting these genes are likely evolutionarily conserved (Figure 4, Table S4).

### 3.3. Analysis of Cis-Acting Elements in ScTCP Promoters and Protein-Protein Interaction Network

**Figure 5:**
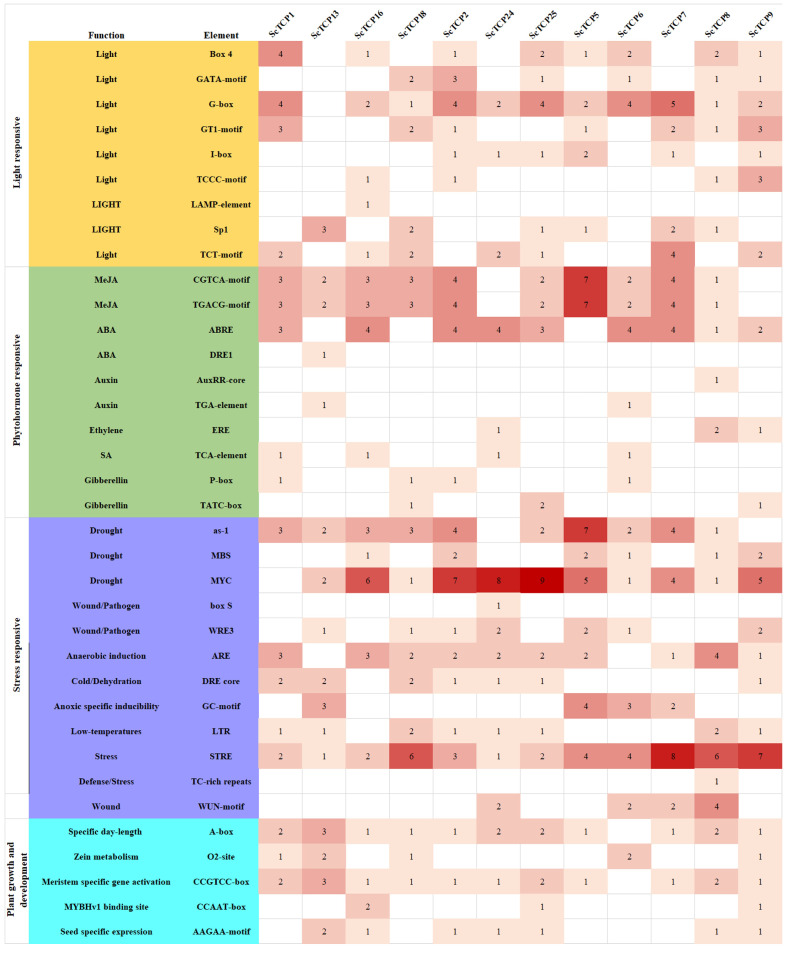
*Cis*-acting elements in *ScTCP* gene promoters, functionally classified into four categories: plant growth and development, hormone response, and light and stress response.

The promoter regions [43] of *ScTCP* genes were found to contain a rich abundance of *cis*-acting elements, which were categorized into four groups based on their enrichment level: hormone-responsive, light-responsive, stress-responsive, and plant growth-related elements. The most enriched elements in a single gene were associated with MeJA-response and drought stress. Stress response elements, such as those for low temperature, drought, and anaerobic conditions, were identified in nearly all *ScTCP* genes. Light (G-BOX), drought (MYC), MeJA, and ABA response elements were found in 91.7% of the genes. Numerous drought-related MYC elements were contained within *ScTCP2*, *ScTCP24*, and *ScTCP25*. All genes, except *ScTCP9* and *ScTCP24*, were found to possess varying numbers of MeJA-responsive elements (*p* < 0.05) (Figure 5, Table S5).

**Figure 6:**
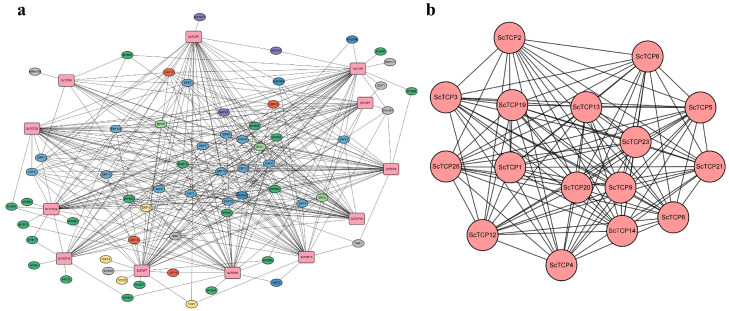
(**a**) Regulatory networks between ScTCPs and potential transcription factors (TFs). Red boxes represent *ScTCP* genes, and ovals represent different TFs. (**b**) Sixteen *ScTCP* genes showing protein–protein interactions with *T. aestivum* homologs.

Furthermore, to explore the regulatory mechanisms of cis-acting elements on the expression of *ScTCP* genes, the PlantTFDB database was used to conduct a systematic analysis of cis-regulatory elements in the promoter regions of all *ScTCP* genes. The results showed that the most abundant transcription factor binding sites were present in *ScTCP5*, whereas the fewest were found in *ScTCP9*. All the *ScTCP* genes contained numerous ERF and MYB TF binding sites, indicating their importance in ScTCP regulatory processes. Concurrently, the promoters of some *TCP* genes (*ScTCP5*, *ScTCP7*, and *ScTCP13*) were found to be bound by others to regulate *ScTCP1* expression (Figure 6a). The proteins of the 16 ScTCPs were predicted to interact at different levels; most interacting proteins were associated with ScTCP1 and ScTCP20, while only one interacting protein was associated with ScTCP8, ScTCP9, and ScTCP12 (*p* < 0.05) (Figure 6b).

### 3.4. Expression Patterns of ScTCP Genes

**Figure 7:**
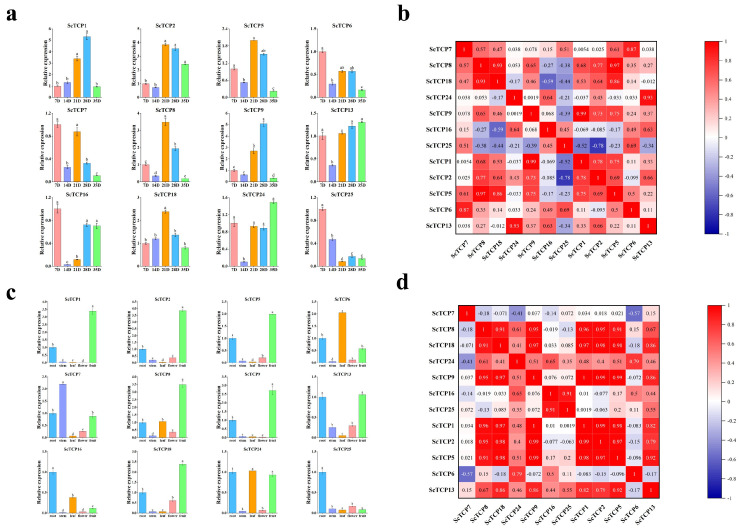
Tissue−specific expression of 12 *ScTCP* genes at different developmental stages. (**a**) qRT-PCR expression profiles of 12 *ScTCP* genes in flower, leaf, root, stem, and fruit tissues. Error bars represent standard errors from three replicates. Lowercase letters indicate significant differences among treatments (α = 0.05, LSD). (**b**) Positive values indicate positive correlations, and negative values indicate negative correlations. Red numbers denote significant correlations (*p* < 0.05). (**c**) qRT−PCR analysis of 12 *ScTCP* genes during fruit development (7, 14, 21, 28, and 35 DPA). Error bars represent standard errors from three replicates. Lowercase letters indicate significant differences among treatments (α = 0.05, LSD). (**d**) Positive values indicate positive correlations, and negative values indicate negative correlations. Red numbers denote significant correlations (*p* < 0.05).

Numerous studies have increasingly highlighted the significance of TCP transcription factors in regulating plant development [44]. To further validate gene functionality, the expression of the *ScTCP* gene was investigated across various tissues by qRT-PCR. A select group of 12 representative genes from the three subfamilies was chosen to examine their expression patterns in roots, stems, leaves, and flowers. As depicted in Figure 7, the *ScTCP* gene demonstrated distinct tissue-specific expression patterns. Most genes exhibited high expression in fruits, while displaying low expression in stems and leaves. Tissue-specific expression being displayed by certain *ScTCP* genes, such as *ScTCP1*, *ScTCP2*, *ScTCP5*, *ScTCP8*, *ScTCP9*, and *ScTCP18*, which exhibited higher expression levels in fruits compared to other tissues. Notably, *ScTCP6* showed peak expression in leaf tissue, while *ScTCP16* and *ScTCP25* displayed the highest expression levels in the root (Figure 7a,b). The high expression of most genes in fruits indicates that the *TCP* gene family may be more related to fruit development (*p* < 0.05).

Further, the 12 genes related to fruit development were selected to explore tissue-specific expression at five different *rye* post-anthesis periods (7 DPA, 14 DPA, 21 DPA, 28 DPA, and 35 DPA). Different expression patterns at different times were shown by the 12 genes (Figure 7c,d). The expression of *ScTCP6*, *ScTCP7*, *ScTCP16*, and *ScTCP25* was observed to gradually decrease with time, while a positive correlation with time was shown by four genes (*ScTCP2*, *ScTCP5*, *ScTCP8*, and *ScTCP18*). The highest expression was observed for *ScTCP13* and *ScTCP24* at 21 DPA, and for at 35 DPA (*p* < 0.05).

### 3.5. Expression Patterns of ScTCP Genes Under Various Treatments

**Figure 8:**
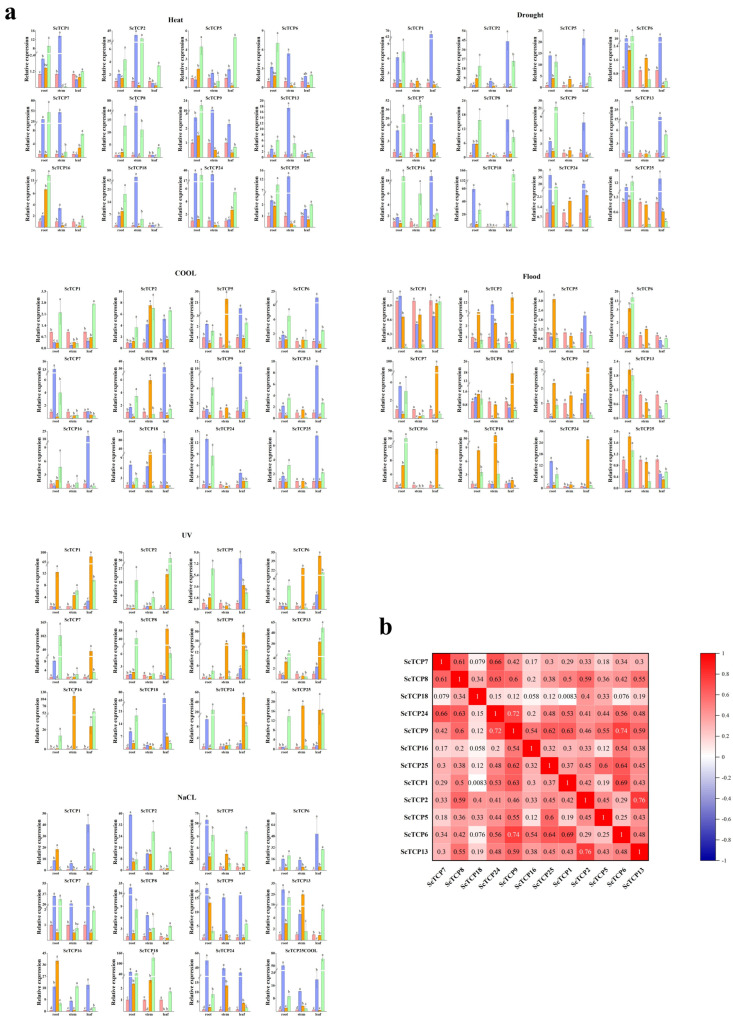
Expression patterns of 12 *TCP* genes under abiotic stresses (UV radiation, flooding, PEG, NaCl, heat, and cold) at the seedling stage. (**a**) qRT−PCR analysis of *TCP* gene expression across different tissues (root, stem, and leaf). Lowercase letters above the bars indicate significant differences among treatments (α = 0.05, LSD). (**b**) Positive values indicate positive correlations, and negative values indicate negative correlations. Red numbers denote significant correlations (*p* < 0.05).

The expression of 12 *ScTCP* genes was examined under six abiotic stress conditions to evaluate their responses to different stresses. Several *ScTCP* genes were significantly upregulated or downregulated under specific stress treatments. Furthermore, the expression patterns of most *ScTCP* genes differed significantly among tissues under the various treatments. For example, cold stress upregulated most *TCP* genes in leaves, whereas heat stress predominantly upregulated TCP genes in roots and stems. Notably, the expression levels of *ScTCP6*, *ScTCP8*, *ScTCP9*, *ScTCP13*, *ScTCP16*, *ScTCP18*, and *ScTCP25* differed among tissues under stress conditions. *ScTCP2*, *ScTCP7*, *ScTCP8*, *ScTCP9*, *ScTCP18*, and *ScTCP24* were significantly upregulated in leaves under flooding stress, particularly at 4 h. *ScTCP6*, *ScTCP9*, *ScTCP13*, *ScTCP16*, *ScTCP24*, *ScTCP26*, and *ScTCP28* were significantly upregulated in roots under drought stress (Figure 8a,b). Meanwhile, most genes also showed significant upregulation under UV and NaCl stresses. *ScTCP6* was significantly upregulated under all six treatments and may therefore represent a potential candidate gene (*p* < 0.05).

**Figure 9:**
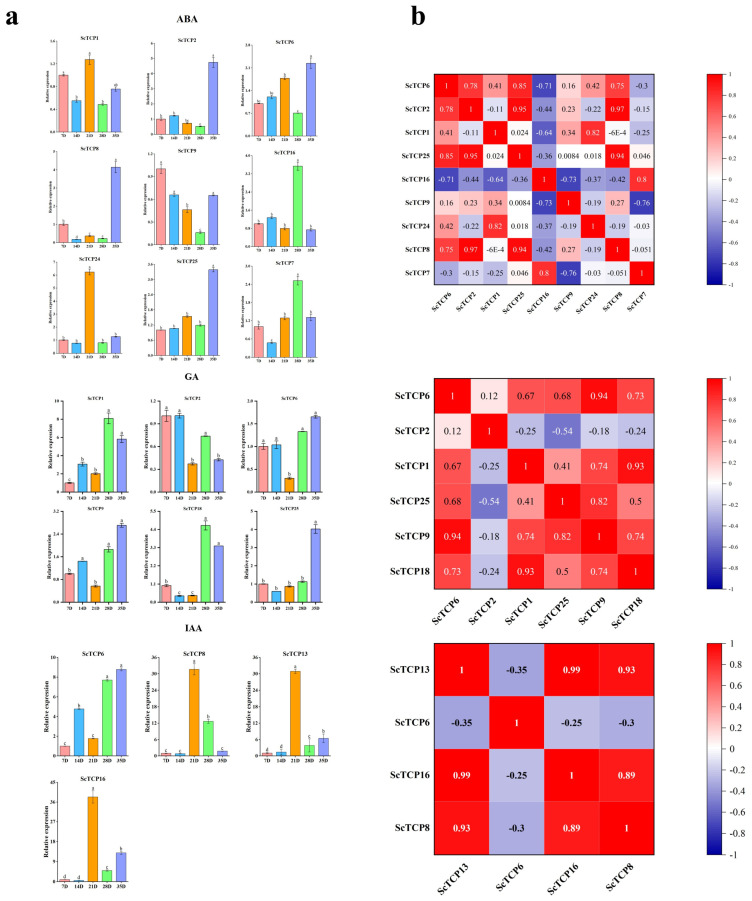
Expression changes in *TCP* genes under three hormone treatments (ABA, IAA, and GA_3_) in rye fruits. (**a**) Expression analysis of the *ScTCP* genes was performed using qRT-PCR. Error bars represent standard errors from three replicates. Lowercase letters above the bars indicate significant differences among treatments (α = 0.05, LSD). (**b**) Positive values indicate positive correlations, and negative values indicate negative correlations. Red numbers denote significant correlations (*p* < 0.05).

In addition, the expression patterns of *ScTCP* genes under ABA, IAA, and GA3 treatments were analyzed to further explore their potential functions. The genes exhibited distinct expression patterns in response to different hormone treatments. Most genes were upregulated under ABA treatment, with the exception of *ScTCP9*, which was downregulated. Under IAA treatment, *ScTCP6*, *ScTCP8*, *ScTCP13*, and *ScTCP16* were significantly upregulated (*p* < 0.05). *ScTCP2* was downregulated under GA treatment (Figure 9a,b). Notably, *ScTCP6* was significantly upregulated in all tissues under both hormone and abiotic stress treatments and therefore should be further investigated.

## 4. Discussion

*Rye* is noted for its high nutritional value and robust adaptability to various abiotic stresses, including cold, drought, and salinity, underscoring its agricultural importance [45,46]. In this study, 26 *TCP* genes (*ScTCP*) were identified in the *rye* genome, a number similar to those in *Arabidopsis* (24) and *rice* (22), indicating a conserved evolutionary pattern. However, a notably higher number of *TCP* genes (66) is possessed by wheat, which is likely attributed to its complex hexaploid genome and consequent expansion events [47,48]. This divergence suggests that gene family expansion is influenced by genome duplication events.

The *ScTCP* genes were categorized into three distinct subfamilies by phylogenetic analysis, a classification consistent with *Arabidopsis* and *rice*, suggesting that TCP families diverged before the separation of dicotyledon and monocot plants. Structural analysis of ScTCP proteins revealed significant variations in protein lengths (ranging from 162 to 461 amino acids). Intriguingly, introns were lacked by most *ScTCP* genes (14); this difference may be attributed to duplication events and different subunit combinations within gene families. Motif 1 was identified as universally present across all TCP proteins by motif analysis, highlighting its potential essential role. Variations in other motifs across the subfamilies indicate functional diversification, likely enabling adaptation to diverse biological processes [49].

Tandem and fragment duplication events are considered important amplification modes in the evolution of plant functional diversity [50]. Segmental duplication events were revealed as the predominant evolutionary mechanism for *TCP* gene family expansion in rye by gene duplication analysis, whereas tandem duplication events were not detected. This study identified only two pairs of fragmentally duplicated genes, namely *ScTCP1*/*ScTCP6* and *ScTCP12*/*ScTCP22*, both of which are located in subfamilies 1 and 3 of the *TCP* gene family. This result suggests that subfamilies 1 and 3 have played a crucial role in the expansion and evolution of the wheat *TCP* gene family. Such band-like duplication events can generate redundant gene copies, providing an initial molecular template for subsequent mutation accumulation and functional differentiation. This process substantially promotes functional diversification and structural complexity within the gene family and lays an important molecular foundation for wheat to resist diverse environmental stresses and adapt to heterogeneous habitats [51,52].

Furthermore, *TCP* genes are divided into three subfamilies across different species, consistent with the previous classification established in *Arabidopsis*. The highest orthology was observed between rye and wheat *TCP* genes, as indicated by collinearity analysis, which aligns with their close evolutionary relationship. Interestingly, collinearity with *rye ScTCP* genes was shared by several species (*O. sativa*, *H. vulgare*, *A. tauschii* Coss, and *Z. mays*). For example, *ScTCP2* is colinear with *AET2Gv20566900.3*/*AT1G30210.1*/*Os07t0152000-01*/*HORVU5Hr1G103400.1*/*TraesARI2A01G274900.1*/*Zm00001d007868_T001*. Seven *ScTCP* genes (*ScTCP1*, *ScTCP6*, *ScTCP8*, *ScTCP12*, *ScTCP15*, *ScTCP21*, and *ScTCP22*) were found to be collinear with *T. aestivum*, suggesting common ancestral origins and potentially conserved biological functions. This linear characteristic provides an important target for the molecular breeding of cereal crops. It can be utilized to facilitate the directional introgression of superior *TCP* genes from rye into wheat, thereby broadening the genetic basis for wheat improvement. In addition, the analysis showed that *TCP* genes contain 10 distinct motifs, with different subfamilies exhibiting similar motif patterns. Notably, *TCP* genes in subfamily 3 contain almost all identified motifs. These results further indicate that *TCP* genes in rye and *T. aestivum* are more closely related and may share a common ancestor.

Promoter analysis is significant for understanding gene regulatory mechanisms, predicting expression levels, and identifying potential regulatory elements [34]. Notably, drought-responsive (AS-1) and MeJA-responsive elements were identified as prevalent, indicating a potential role for *TCP* genes in stress adaptation. All *ScTCP* promoters contain the drought-related element AS-1, which can be bound by transcription factors to regulate stress-responsive genes, thereby enhancing plant adaptability (drought, salt stress, and low temperature) [53]. Besides *ScTCP9* and *ScTCP24*, varying numbers of MeJA-responsive elements were present in all promoters. These MeJA response elements [54] bind transcription factors to improve stress adaptability when the MeJA signaling pathway is activated by stresses. The largest number of TFS was found in *ScTCP5*, while the fewest were found in *ScTCP9*, indicating that ScTCP5 proteins are composed of multiple structural or functional domains. Therefore, ScTCP5 proteins can interact with numerous different proteins for multiple signal transduction or functional modeling. It should be noted that the conclusions of this study are based solely on bioinformatics analyses. Further experimental validation of gene functions and a more comprehensive elucidation of the related regulatory mechanisms will be required through molecular biology experiments in future studies.

Distinct tissue-specific and developmental-stage-specific expression patterns for *ScTCP* genes were revealed by expression profiling. Most of the 12 representative *TCP* genes were highly expressed in fruits, highlighting their potential roles in the regulation of this critical agronomic trait. The expressions of six genes (*ScTCP1*, *ScTCP2*, *ScTCP5*, *ScTCP8*, *ScTCP9*, and *ScTCP18*) were found to be significantly higher in fruits compared to other tissues. Previous studies showed that in *Arabidopsis*, overexpressing *TCP15* leads to smaller fruits, whereas overexpressing *TCP2* and *TCP4* alters fruit morphology significantly [55]. Given that *ScTCP1*, *ScTCP2*, and *ScTCP5* belong to subfamily 3 and share homology with *AtTCP2*, these genes are considered potentially crucial regulators of fruit development in rye [56]. The rice gene *OsTCP1*, which is the homolog of *ScTCP5* in rye, can regulate the development process of anther through finely controlling the biosynthesis pathway of jasmonic acid (JA) [57]. Furthermore, we found that most *ScTCP* genes displayed significant differential expression patterns under various abiotic stress conditions. For example, most genes were significantly upregulated in stems and roots under cold stress. Under heat stress, increased gene expression was predominantly observed in root tissues, with similar expression patterns being shown by some genes under both cold and heat stress. Interestingly, opposite expression tendencies between leaf and root tissues were exhibited by *ScTCP6*, *ScTCP8*, *ScTCP9*, *ScTCP13*, *ScTCP16*, *ScTCP18*, and *ScTCP25*, probably because tissue-specific physiological and molecular response pathways are triggered by cold and heat stress. The barley *HvTB1* (also known as VULGARE SIX-ROWED spike 5, *VRS5*) gene is homologous to the rye *ScTCP6* gene. Its expression can inhibit the occurrence of tillers (side branches) in the plant and the development process of the grains [31]. Leaves, which are the main sites for photosynthesis and gas exchange, as well as the primary source and consumption site of nutrients and water, possess strong sensitivity and adjustment ability to environmental stress. The most obvious responses to flooding and UV stress were observed in leaves. Conversely, under salt stress, significant responses were predominantly observed in roots, as roots are directly exposed to a high salinity soil environment and assume important functions such as salt uptake, ion balance regulation, cell protection, and rhizosphere microbial interaction. Previous studies showed that WRI1 attenuates *GH3.3* expression through its interaction with TCP20 in *Arabidopsis*, thereby regulating plant development through auxin regulation [58]. In this study, *ScTCP8*, *ScTCP13*, and *ScTCP16* are most significantly upregulated after IAA treatment, suggesting they might be critical in auxin synthesis pathways in rye.

## 5. Conclusions

This study provides a comprehensive characterization of the *TCP* transcription factor family in rye, encompassing gene identification, structural and evolutionary analyses, regulatory element profiling, and expression patterns under various stress conditions and hormonal treatments. Most genes showed significant differential expression in response to these factors, confirming the involvement of the *TCP* gene family in rye growth and development. Particularly, different stress treatments significantly upregulated *ScTCP6*, highlighting its potential role in enhancing rye stress resistance through transgenic approaches or its application as a molecular marker in breeding programs. These findings provide an important theoretical basis for molecular breeding strategies based on the *ScTCP* gene family.

## Data Availability

The whole genome sequence information of rye was obtained from the Ensembl genome website (http://ensemblgenomes.org/). In the experiment, the rye material used was provided and licensed by Yu Fan from Guizhou University. The datasets supporting the conclusions of this study are included in the article and its additional files.

## Supplementary Materials

The following supporting information can be downloaded at: https://www.mdpi.com/article/10.3390/cimb48030266/s1, Figure S1: Multiple alignment of ScTCP and selected bHLH domain amino acid sequences; Figure S2: Conserved motif and gene structure analysis of 26 *ScTCP* genes in the phylogenetic tree; Table S1: List of 26 *ScTCP* genes identified in this study; Table S2: Tandem duplication events of *ScTCP* genes; Table S3: Conserved motif distribution in TCP proteins across eight species; Table S4: One-to-one orthologous relationships between *Secale cereale* and eight other species; Table S5: Cis-regulatory elements in *TCP* gene promoter regions; Table S6: Primer sequences used in this study.

## Abbreviations

The following abbreviations are used in this manuscript:

*AtTC**P*	*Arabidopsis thaliana TCP*
DPA	Days post anthesis
HMM	Hidden Markov Model
LG	Linkage group
pI	Isoelectric point
*q*RT-PCR	Quantitative real-time polymerase chain reaction
*ScTCP*	*Secale cereale* L. *TCP*
*TCP*	Teosinte branched1/cycloidea/proliferating cell factor
