# Supplementary material for: Genome-Wide Analysis Identifies ScTCP6 as a Stress Responsive Gene in Rye
Source: Curr Issues Mol Biol. 2026 Mar 2;48(3):266. doi: 10.3390/cimb48030266 (PMC13024843; doi:10.3390/cimb48030266)
Supplement: Supplementary file 1 [file cimb-48-00266-s001.zip › Supplementary Figures.pdf]

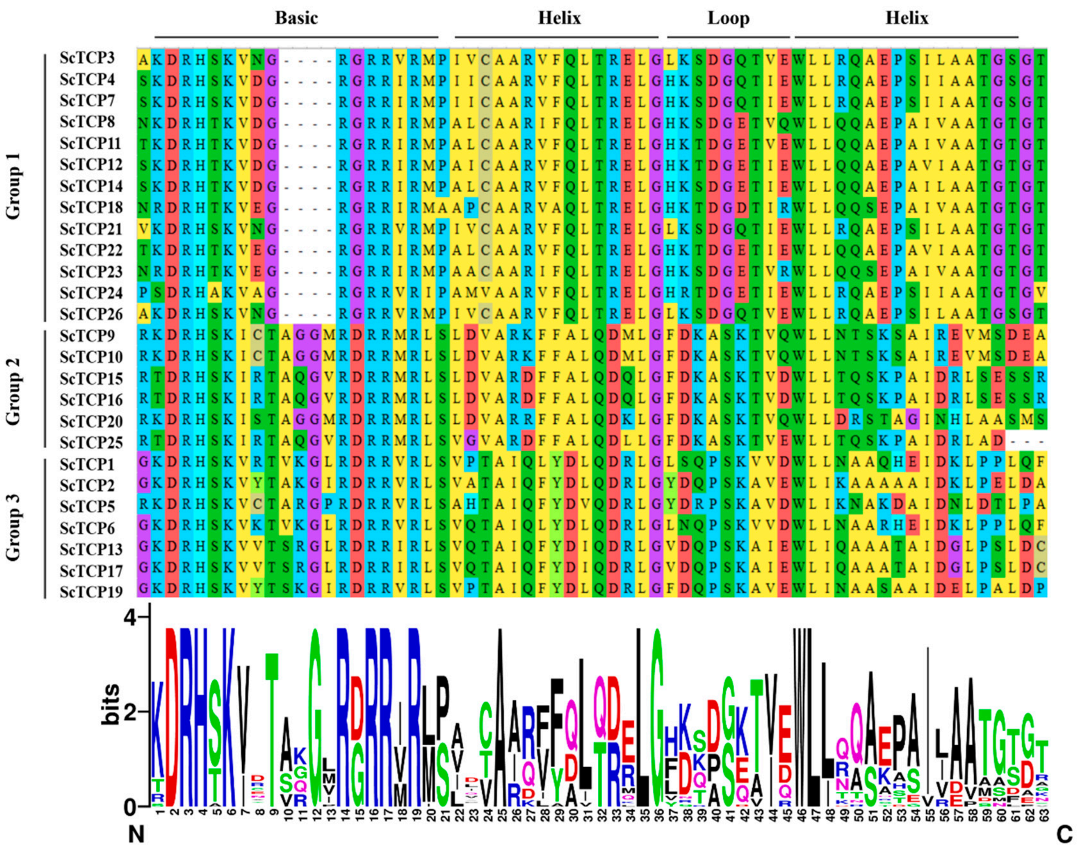

Figure S1: Alignment of multiple ScTCP and select BHLH domain amino acid sequences

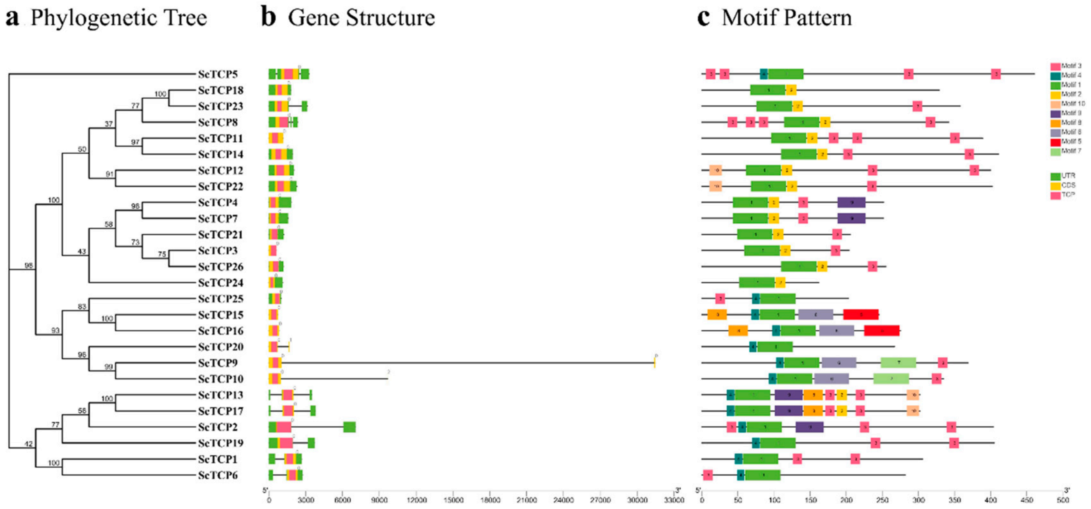

Figure S2: Analysis of conserved motifs and gene structures in the phylogenetic tree of 26 *ScTCP* genes.
